# Melanin for Photoprotection and Hair Coloration in the Emerging Era of Nanocosmetics

**DOI:** 10.3390/ijms25115862

**Published:** 2024-05-28

**Authors:** Arianna Menichetti, Dario Mordini, Silvia Vicenzi, Marco Montalti

**Affiliations:** 1 Department of Chemistry “Giacomo Ciamician”, University of Bologna, Via Selmi 2, 40126 Bologna, Italy; arianna.menichetti2@unibo.it (A.M.); dario.mordini2@unibo.it (D.M.); silvia.vicenzi3@studio.unibo.it (S.V.); 2 Department of Chemistry “Giacomo Ciamician”, University of Bologna, Tecnopolo di Rimini, Via Dario Campana 71, 47921 Rimini, Italy

**Keywords:** melanin, polydopamine, allomelanin, nanoparticles, ROS, biocompatibility, toxicity, nanotoxicity, biomimetic, dopamine

## Abstract

Nanotechnology is revolutionizing fields of high social and economic impact. such as human health preservation, energy conversion and storage, environmental decontamination, and art restoration. However, the possible global-scale application of nanomaterials is raising increasing concerns, mostly related to the possible toxicity of materials at the nanoscale. The possibility of using nanomaterials in cosmetics, and hence in products aimed to be applied directly to the human body, even just externally, is strongly debated. Preoccupation arises especially from the consideration that nanomaterials are mostly of synthetic origin, and hence are often seen as “artificial” and their effects as unpredictable. Melanin, in this framework, is a unique material since in nature it plays important roles that specific cosmetics are aimed to cover, such as photoprotection and hair and skin coloration. Moreover, melanin is mostly present in nature in the form of nanoparticles, as is clearly observable in the ink of some animals, like cuttlefish. Moreover, artificial melanin nanoparticles share the same high biocompatibility of the natural ones and the same unique chemical and photochemical properties. Melanin is hence a natural nanocosmetic agent, but its actual application in cosmetics is still under development, also because of regulatory issues. Here, we critically discuss the most recent examples of the application of natural and biomimetic melanin to cosmetics and highlight the requirements and future steps that would improve melanin-based cosmetics in the view of future applications in the everyday market.

## 1. Introduction

Nanomaterials and nanotechnology are revolutionizing fields of high social and economic impact, such as medicine [1,2,3,4,5,6], energy conversion and storage [7,8,9,10], and environmental remediation [11,12,13]. Nevertheless, public opinion is still worried by the potential negative effects of nanomaterials on human health and the environment, and legislation regarding the large-scale use of nanomaterials is still partially undefined [13]. Cosmetic formulations have contained, since their ancient origin, nanostructures such as micelles [14], nanodroplets [15], nanosized sunscreens [16], and pigments and fillers [17,18] and they can be considered as dispersions often containing nanosized units. These nanostructures result either from supramolecular (non-covalent) self-assembly (bottom-up processes) or are the result of the disaggregation of bulk materials (top-down process) that are present in the final product [19]. While these spontaneously formed nanostructure are generally tolerated by customers, pre-formed nanomaterials incorporated in cosmetics as such still raise concerns because they are considered “unnatural” and hence potentially dangerous [20,21]. Here, we demonstrate that, in this framework, melanin presents unusual features. Melanin exists in nature already in the form of nanoparticles in the ink of several animals, like cuttlefish, and as nano-aggregates in the human skin and hair [22]. Melanin nanostructures play important roles in nature, including photoprotection and coloration [23]. Their biomimetic copies, such as polydopamine (PDA), have been demonstrated to be almost completely identical to the natural ones, to the point that human skin cells (keratinocytes) treat PDA nanoparticles (NPs) exactly as bio-produced melanin [24]. In this review, we provide an overview of recent systems based on melanin NPs, investigated for their applications in hair coloration and photoprotection. Moreover, we report the main critical aspects that, at the moment, limit the application of melanin-based cosmetics. Indeed, once the necessary investigations and improvements are performed, and thanks to their high biocompatibility and their unique optical and electronic properties, natural and biomimetic melanin nanoparticles can pave the road to the advent of the new science of nanocosmetics [25].

## 2. Chemistry of Natural and Biomimetic Melanin

Melanin represents a family of pigments present in many animal and vegetal species, where they play important roles in coloration and photoprotection [23].

**Figure 1:**
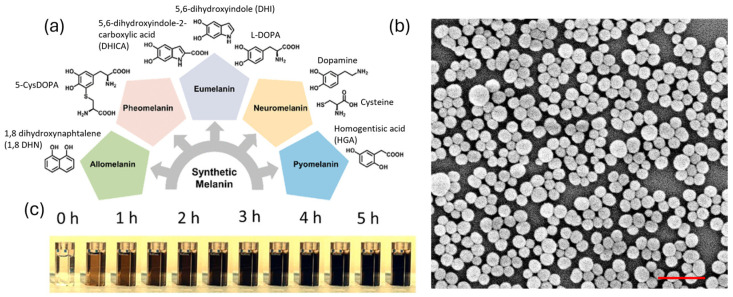
(**a**) Classification of different kinds of melanin based on their molecular precursor, from ref. [28]. (**b**) SEM image of sepia ink. Scalebar is 1000 nm. (**c**) Optical images of cuvettes containing dopamine (0 h) during oxidation by atmospheric oxygen. Each cuvette represents the reaction mixture at successive 30 min time periods from the beginning of the reaction (time 0 h). The change in color, which goes from light to dark brown, denotes the progressive formation of PDA NPs. Adapted with permission from ref. [27]. Copyright 2022, American Chemical Society.

Melanin is a polymeric species which arises from the oxidation and polymerization of one or more molecular precursors. Since the actual chemical composition and structure of the different kinds of melanin are largely unknown [26,27], the most convenient approach to their classification is still based on the monomer they originated from [28] (Figure 1a). The biosynthetic path to melanin involves oxidation performed by enzymes. In the case of “eumelanin”, the dark pigment present in human hair and skin and the most studied natural melanin form, the molecular precursor is tyrosine, which is first oxidized to L-dopa by tyrosinase. Further oxidation leads to the formation of eumelanin. Copolymerization with cysteine, on the other hand, can lead to the formation of pheomelanin, the orange–red pigment present in blond–red human hair [29,30,31]. Besides their similar origin, eumelanin and pheomelanin are so different that while eumelanin is known to be photoprotective, pheomelanin is considered, on the contrary, phototoxic.

Although different kinds of melanin are largely present in nature, their extraction for technological applications is often inconvenient, since it is typically cost and time demanding and poorly efficient. Artificial synthesis of melanin, on the other hand, is very effective and, most importantly, very versatile. Oxidation of the precursors can be achieved using safe, low-cost, and widely available oxidants like atmospheric molecular oxygen. This is the case of the synthesis of polydopamine (PDA), considered the artificial biomimetic copy of eumelanin and obtainable from the oxidation/polymerization of dopamine in slightly alkaline conditions, in aqueous solution, in the presence of atmospheric oxygen at room temperature. This synthetic process is highly environmentally friendly and leads to highly biocompatible PDA nanoparticles (Figure 1b,c). Additionally, the process is highly versatile, thanks to the presence of different functional groups both in DA and in PDA. For this reason, PDA has found applications in fields of high social and economic relevance, such as medicine [32,33,34,35], energy conversion and storage [36], and environmental remediation [37]. The popularity of PDA is demonstrated by the huge number of scientific papers dealing with this material published in the last five years (2020–2024), which totaled more than 8000 at the end of April 2024, according to Web of Science. Besides this, recently some researchers, and in particular the Gianneschi group [38,39], have demonstrated that melanin obtained by oxidation/polymerization of 1,8 dihydroxynaphthalene, as a biomimetic copy of natural allomelanin, presents unique chemical properties that could make these NPs an advantageous alternative to PDA for several technological applications. This point will be discussed more in detail in the next section.

## 3. Properties of Melanin

The most typical features of eumelanin and PDA are their unique biocompatibility and their broad light adsorption band that ranges from the UV to the NIR and which is responsible for the dark color of these nanomaterials [40,41]. Adsorption is indeed only one of the two components of the extinction of light by PDA since, in the form of NPs, this material is also able to scatter light. The actual origin of the broad band absorption of eumelanin and PDA NPs is still debated, as is as their actual chemical composition. For this reason, these materials have been object of detailed investigation via ultra-fast transient spectroscopy (UFTS) aimed at understanding their interactions with light. Despite the different conclusions reached by different researchers, the presence of different chromophores in these forms of melanin, and in particular of di-hydroxyindole (DHI) units and their oxidized quinone forms, is almost univocally accepted [42]. Hence, optical transitions could be either localized on a large variety of chromophores, each responsible for the absorption in a specific optical range, or involve optical electron transfer processes between different chromophores [43,44,45]. The former hypothesis justifies the typical hole-burning, wavelength-dependent behavior observed for eumelanin [43], while the latter is consistent with the appearance of less excitation wavelength-dependent broad transient absorption [44]. In any case, excited-state deactivation of eumelanin is generally a fast process that occurs in the picosecond time scale [46]. Fast excitation energy dissipation is totally compatible with the high photoprotective action of eumelanin, since it suggests that the absence of long-living excited states prevents any photochemical reactivity and hence any phototoxicity. Moreover, the observation that deactivation occurs through a non-radiative path justifies the wide application of these materials as photothermal agents (PA) and hence as materials able to convert efficiently absorbed light (especially in the red/NIR region) into heat [47,48]. Nevertheless, it is well known that eumelanin presents some degree of photoreactivity. Photo-oxidation of eumelanin has been reported by several authors as a process involved in Immediate Pigment Darkening (IPD) in human skin [49]. Recently, the possible use of PDA as a photosensitizer (PS) for light-induced polymerization of some vinyl monomers has also been proposed [50]. From the photochemical point of view, the chemical reaction of short-living excited states with other diffusing molecules like oxygen is unexpected, and we believe the photoreactivity of eumelanin and PDA deserves further investigation. Regarding the excited-stated deactivation path, fluorescence of PDA samples has also been reported [51]. According to our findings, this fluorescence is due mostly to PDA NP fragments, rather than to the NPs themselves [41]. Generally, the origin of the fluorescence of PDA needs further study.

The photoprotective action of eumelanin and PDA does not arise only from their abilities of absorbing and scattering light but is also the result of their efficient antioxidant activity. PDA was demonstrated to be an efficient radical scavenger able to limit the formation of reactive oxygen species (ROS), which are considered to be at the base of the indirect damage of skin due to light and involved in skin aging [52]. In this context, artificial allomelanin was demonstrated to be a much more effective antioxidant agent than PDA [39]. Thanks to this feature, allomelanin may in the future substitute PDA in all applications which take advantage of antioxidant activity [53].

## 4. Nanomaterials in Cosmetics

Despite the application of nanotechnology to cosmetics still being a critical issue [54], cosmetic products contain, in most formulations, nanosized objects. A typical example is micelles, which spontaneously form from the self-assembly of surfactants used as emulsifier or dispersant agents in cosmetic formulations [14]. Micelles are NPs with typical diameters ranging from a few to tens of nanometers and are often used, because of their high mono-dispersity, as templates for the synthesis of other NPs. Microemulsions used in cosmetic products are unique emulsions that present thermodynamic stability and contain droplets with sizes of a few tens of nanometers [15,55]. Inorganic sunscreens like titanium and zinc oxides filter solar light by scattering and, to give a Rayleigh-like wavelength-dependent response and enhance protection from the UV spectrum, they need to be present in sizes of a few tens of nanometers [15]. In this way, these particles look transparent and without color in the visible spectrum, while micrometric particles show Mie scattering and a whitish appearance. Recently, the use of silver nanoparticles in cosmetics had been proposed, in virtue of their anti-bacterial activity [56]. What is the difference between all these kinds of NPs? According to the regulations of the European Commission for cosmetic products: “nanomaterial means an insoluble or bio-persistent and intentionally manufactured material with one or more external dimensions, or an internal structure, on the scale from 1 to 100 nm” [57]. According to this definition, micelles and titania NPs are classified very differently, and presently only 29 nanomaterials (three as colorants, four as UV filters, and 22 with other functions) are allowed in cosmetics, including silver NPs (Table 1).

As a condition, the use of these nanomaterials in cosmetics is possible only if their presence in nano-form is declared by the producer and notified to the Cosmetic Products Notification Portal (CPNP) six months before placing the product on the market. Melanin is not included in the list. In particular, melanin is present among the permitted cosmetic ingredients only for the China Food & Drug Administration (CFDA) [59], while the Food and Drug administration (FDA) [60], Canada’s cosmetic regulations [61], and the Association of Southeast Asian Nations (ASEAN) [62] do not consider melanin either in their permitted or in their prohibited ingredients, despite melanin already being present as nanoparticles in human skin and human hair [22]. What will be the future of melanin-like nanomaterials in cosmetics?

## 5. Melanin for Photoprotection

**Figure 2:**
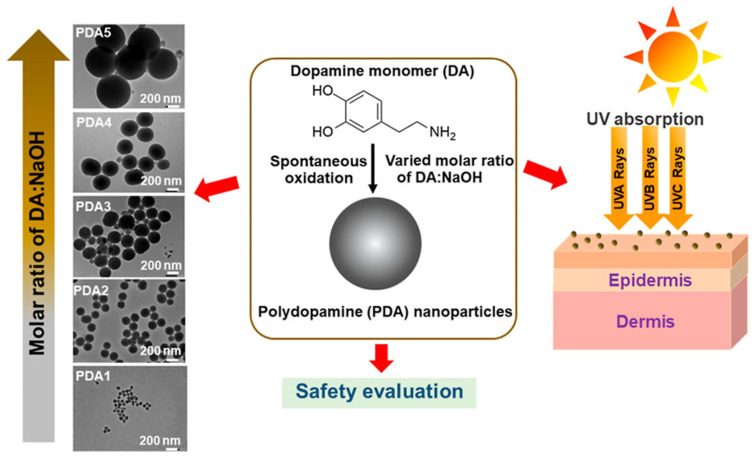
PDA NP production (PDA1, PDA2, PDA3, PDA4, and PDA5) as solar filters. Reprinted with permission from ref. [76]. Copyright 2022, American Chemical Society.

The exposure of our skin to UV radiation has numerous effects in our bodies, such as the synthesis of vitamin D and endorphins, tanning, and immunoregulation [63]. However, overexposure to UV radiation has been reported to induce DNA damage and skin cancer [64,65]. The strategy that the human organism adopts to contrast the negative effects of UV radiation is the production of melanin as a photoprotective agent. The synthesis of melanin is stimulated by sunlight and occurs in melanocytes, cells located in the *stratum basale* of the epidermis [66,67]. This process is responsible for our skin pigmentation, and the produced melanin shields from UV radiation by means of its very broad UV–vis absorption band and radical scavenging action [68]. However, natural skin pigmentation has resulted to be not sufficient to prevent UV-caused damage and needs to be integrated with synthetic solar filters. Sunscreens are usually multicomponent systems obtained by the mixture of one or more solar filters and various stabilizers [69]. There are mainly two categories of solar filters, named inorganic and organic solar filters. Inorganic solar filters are usually based on particulates, among which the most common materials are nanosized titanium dioxide and zinc dioxide [70]. Organic solar filters comprise aminobenzoic and salicylic acid derivatives, triazines, and benzimidazole and benzotriazole derivatives [71]. Both inorganic and organic filters exert their function by an efficient absorbance of UVA (λ = 400–315 nm) and UVB (λ = 315–280 nm) light. Nevertheless, there are some concerns about their possibility to penetrate through the skin and their production of reactive oxygen species (ROS) [72,73]. For these reasons, solar filters are often coupled with antioxidants or embedded in matrices that prevent skin penetration [74,75]. However, having a complex formulation is sometimes not beneficial, since the combination of the components could lead to a decrease of filter efficiency because of their chemical or photochemical interactions. In this area of optics, research is focusing on melanin-based sunscreens, mimicking the role that it has in our skin. Indeed, melanin, other than being an excellent photoprotective agent, has also high biocompatibility and antioxidant activity, summarizing all the requirements of a proper solar filter. PDA NPs are usually studied as solar filters, as described in the paper of Supanakorn and co-workers [76]. Their nanoparticles were produced through spontaneous oxidation reactions between a dopamine monomer (DA) and sodium hydroxide (NaOH) (Figure 2).

**Figure 3:**
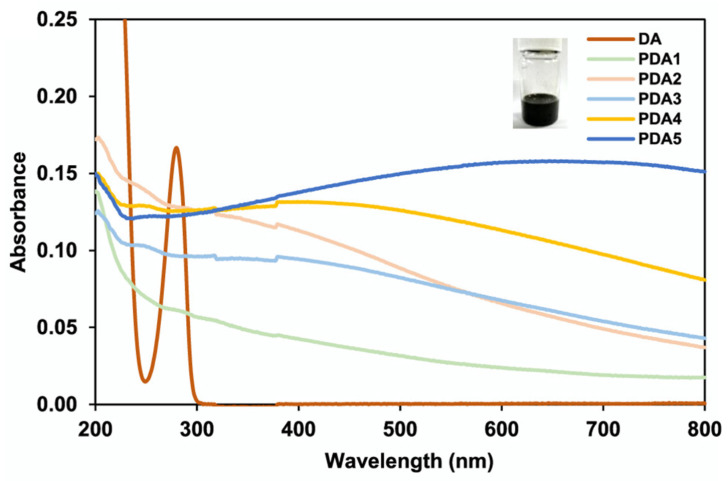
UV−vis absorption spectra of PDA nanoparticles and DA. Reprinted with permission from ref. [76]. Copyright 2022, American Chemical Society.

By varying the molar ratio of DA/NaOH, they were able to control the size of the nanoparticles. The molar ratio was varied from 1:1 to 1:0.8, 1:0.6, 1:0.4, and 1:0.2 to prepare different sizes of spherical nanoparticles, denoted as PDA1, PDA2, PDA3, PDA4, and PDA5, with sizes ranging from 59.5 to 659.1 nm. The PDA NPs presented very good stability in an aqueous medium in different pH conditions (pH > 3.4) and were stable for three months, even when stored at 50 °C. UV–vis absorption spectra (Figure 3) showed that PDA2 presented the highest UVB absorption (290−320 nm) and a sun protection factor boosted by approximately 50% compared to that of the original base formulation. 

Further evaluation through in vitro studies was conducted to define their photoprotective abilities. As a result, HaCaT cells treated with PDA 1–5 nanoparticles prior to UV exposure showed significantly reduced membrane damage in a dose-dependent manner and considerable reduction in ROS. Notably, PDA1, PDA2, and PDA3 also improved cell viability after UVA exposure. Cytotoxicity in vitro studies using HaCaT cells suggested good biocompatibility of the PDA nanoparticles, due to their melanin-mimicking nature. These findings provided a promising approach for synthesizing PDA nanoparticles as safe and effective sunscreen boosters. Demonstration of the efficient photoprotection of melanin-like materials was also reported in the paper of Liberti et al., in which a melanin-like polymer’s photoprotective performance was evaluated [77]. The polymer was obtained by the polymerization of the methyl ester of DHICA (MeDHICA-melanin). DHICA (5,6-dihydroxyindole-2-carboxylic acid) is one of the melanin precursors, and its modification to MeDHICA was performed to increase MeDHICA-melanin’s solubility in organic solvents, a useful aspect for its potential embedment in dermocosmetics. Indeed, MeDHICA resulted to be soluble in water-miscible organic solvents and it also had a good stability upon oxidation and solar light exposure. MeDHICA-melanin’s biocompatibility and photoprotective activity were tested by incubation in keratinocyte HaCaT cells. Interestingly, other than showing no cytotoxicity, MeDHICA-melanin reduced oxidative stress related to UV light irradiation. Indeed, ROS and lipid peroxidation were significatively reduced, and glutathione levels, whose decrease is correlated with oxidative stress, remained unaltered under irradiation. 

PDA NPs have also been combined with other polymers or embedded in scaffolds to improve their performance and their stability, preventing potential skin penetration. For example, Li et al.’s study involved the development of a bionic photoprotective CS-SA-PDA nanosheet, which showed great potential in terms of photoprotection and photostability [78]. The biomimetic design incorporated sinapic acid (SA), a plant polyphenol that naturally shields against UV rays. Chitosan (CS), a natural polymer, acted as a connecting arm, enhancing the material’s photostability and reducing PDA permeability. As previously mentioned, PDA mimics eumelanin’s light-absorption properties, improving UVA light shielding efficiency and increasing thermal stability, ultimately resulting in better photoprotective performance. In terms of UV-shielding results, the combination of SA and PDA showed a synergistic effect and could render broad-spectrum protection properties. In vivo anti-UV testing was carried out and proved the efficient photoprotective ability of the new material. The treated skin exposed to UV indeed showed no damage and resembled normal skin. CS-SA-PDA possessed excellent skin safety: cell viability decreased slightly with increasing nanosheet concentration but remained over 80%, indicating no apparent biological toxicity. Furthermore, the photoprotective layer exhibited ROS scavenging activity. CS-SA-PDA effectively eliminated photo-induced ROS and, in turn, inhibited chronic photodamage, thus improving the biosafety of the sunscreen. The combination of all these features makes the new biomimetic material ideal for skin photoprotection. Li et al. reported a composite system with hollow PDA NPs embedded in a cross-linked hydrogel formed by chitosan (CS), hyaluronic acid (HA), and β-glycerophosphate (β-GP) to obtain a photoprotective system for sunscreen application [79]. In particular, the use of a hydrogel nanocomposite aimed to find a proper dispersion media for application in sunscreens and to prevent penetration of the NPs through the skin. Indeed, first the biocompatibility of the system was evaluated on NIH-3T3 cells, reporting more than the 80% of viability after 24 h of incubation. Secondly, skin resistance to penetration was tested on porcine skin sections. The nanocomposite hydrogel, compared to bare PDA NPs, showed almost no penetration in the skin, confirming the hydrogel’s protective action and good water resistance, denoting long term stability. The photoprotective ability was evaluated (UVA/UVB ratio), as were sun protection factor (SPF—almost 34% for a PDA NPs content of 100 g/L) and phototoxicity. Compared to the commercial sunscreen TiO_2_, all the tests confirmed a higher shielding ability of the hydrogel nanocomposite based on hollow PDA NPs. Moreover, the choice of having hollow PDA NPs rather than PDA NPs is favorable for the lighter color of the sunscreen, which is more easily applicable to the skin’s surface.

## 6. Melanin for Hair Coloration

### 6.1. Introduction

**Figure 4:**
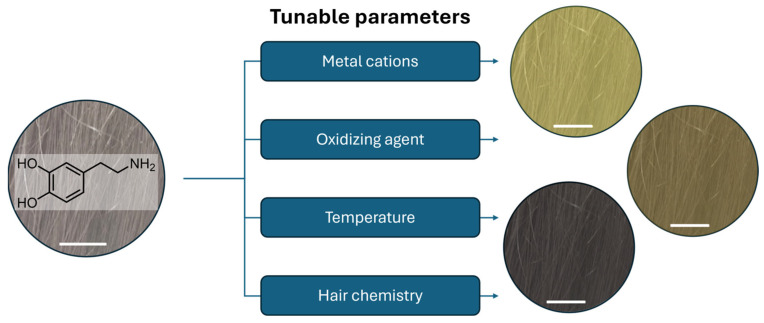
Schematic representation of the list of parameters that can play a role in the deposition of PDA on grey human hair and their effect on the tunability of hair color (from top to bottom: blonde, brown, and black), scale bar 1 cm.

In the following section, we will discuss the employment of melanin-like materials in another crucial area of the cosmetic market: hair coloration. Indeed, we are going to present the state of the art in the application of PDA in this field and we are going to focus on the different approaches researchers have followed to bind these biomimetic pigments to the hair shaft, trying to highlight analogies, differences, and the pros and cons of tuning different parameters, as schematized in Figure 4. To begin with, we are going to introduce the topic, then we will move on to the chemical approaches to dying the hair, and finally we will present the biotechnological approaches.

Hair care has walked with human beings since the dawn of time; indeed, hair beauty has interested every society all over the world, without exclusions related to status, gender, culture, religion, and nationality. In fact, this phenomenon is timeless: archeological finds attest to the cosmetical use of dyeing herbs in ancient Egyptian society [80], whereas nowadays hair care products are advertised on LED screens along the streets of modern metropolises.

To best introduce the topic, it is better to have a dive deep through the biology of hair pigmentation. Color plays crucial roles in the animal kingdom. Indeed, the mantle of pigmentation has been engineered to cover a wide range of needs: it is well known that some animals blend in with their surroundings due to the color of their coat, while others exploit it as a silent form of communication; for example, peacocks exploit it as a mating technique [81]. Among all bio-pigments, melanin is the main one responsible for the pigmentation and structural coloration of feathers and hairs [82,83]. In this context, human beings and primates are not excluded; in fact, melanin is also responsible for the coloration of hair in mammals [84,85]. The natural pigmentation of human hair is determined by the chemical composition and the concentration of melanosomes in the cuticle, cortex, and medulla of the hair fibers. Among human beings, melanosomes can contain different amount of eumelanin and pheomelanin. The former is responsible for black–brown colorations, while the latter confers to hair tresses yellow–reddish nuances [86]. Melanin is bio-synthetized into membrane-enclosed melanosomes by specialized cells (melanocytes) in the hair follicle during the growing phase. In the early stages, melanosomes are mainly composed of proteins, called melanoproteins. As the melanosomes mature, they are transferred from the melanocytes into adjacent keratinocytes, which embed them in a matrix of keratin-associated proteins. Thus, as hair grows, it acquires its color, lasting throughout the lifetime of the hair. As each of us will experience in their life, hair pigmentation does not last forever. Aging, lifestyle, chronic diseases, psychological stress, and photothermal damage are among the main causes of hair greying [87,88,89,90]. If we consider that the World Health Organization (WHO) has estimated that by 2050, the world’s population of people aged 60 years and older will reach 2.1 billion, we can expect that these issues will escalate in the near future. To face this problem, over time, people have developed several solutions, like the use of wigs or hair dyeing. In ancient times, the formulations of hair dye were mainly based on phytochemical products, while with the advent of chemical industries, synthetic dyes quickly became the most popular way to change the color of hair [91]. Despite the expected development in the hair dyeing market, current commercial hair dyeing formulations suffer from several drawbacks. Indeed, in the actual technologies, the use of harsh conditions (like strong alkaline/oxidative solvents) and allergenic dyes is expected. Hence, the application of them on the scalp can bring about the development of adverse reactions like inflammation, dandruff, and dermatitis [92,93]. Regarding this, the use of more biocompatible dyes could be a promising solution to face this problem. Melanin, and in particular PDA, seems best suited for this role: it shows high biocompatibility and antioxidant and photoprotective properties [52]. In addition, PDA NPs are depicted as synthetic analogues of naturally occurring melanosomes [24], which are responsible for human hair pigmentation; indeed, by tuning the physical–chemical properties of synthetic PDA, is possible to mimic every shade of natural hair color. From this premise, it should not surprise that scientists have tried to use synthetic melanin as biomimetic dye. The first attempts to deposit melanin-like materials on hair fibers to restore their coloration go back to some decades ago: Brown et al. exploited the metal-induced oxidation of dihydroxyindole (DHI), one of the precursors of human melanin, to give rise to a new method to color human hair [94]. The idea was to biomimic the biochemical action of tyrosinase, a metalloenzyme that regulates the physiological oxidation of tyrosine into eumelanin. The innovative method was designed in a two-step process, in which the former was devoted to a pre-treatment of the hair tresses with a Cu(II) ion-doped shampoo, while in the latter step the samples were treated with an aqueous solution of DHI. The protocol guaranteed the fast deposition of a brown–black dye on the periphery of the hairs within 10 min. Interestingly, the authors claimed the possibility to tune the color shade by changing the chemical nature of the involved metal cations. Furthermore, in 1997, Brown et al. updated the previous study, exploring the pigmentation capability of other chemical precursors and oxidating agents [95]. In fact, they aimed to pave new routes to the synthesis of new eumelanin-like and pheomelanin-like materials. Basically, they exploited the oxidation of dihydroxyphenylalanine (DOPA) into DHI and dihydroxyindole-2-carboxylic acid (DHICA), using potassium ferricyanide in a buffered solution, to induce the formation of deep-black dyes, while lighter colors, such as brown, were obtained by starting from less-reactive chemicals, like DOPA methyl ester or adrenaline. In the same work, yellow–reddish shades, analogous to the ones of pheomelanin, were reached through the oxidation of cysteinldopa analogs and hydroxybenzothiazine, triggered by sodium iodate and sodium periodate. Despite this promising attempt, melanin-based materials have never been employed in the hair color market. Indeed, even if the exceptional properties of melanin were well known at that time, the processability of these biomaterials was still challenging, making their application difficult. 

### 6.2. PDA in Hair Coloration

**Figure 5:**
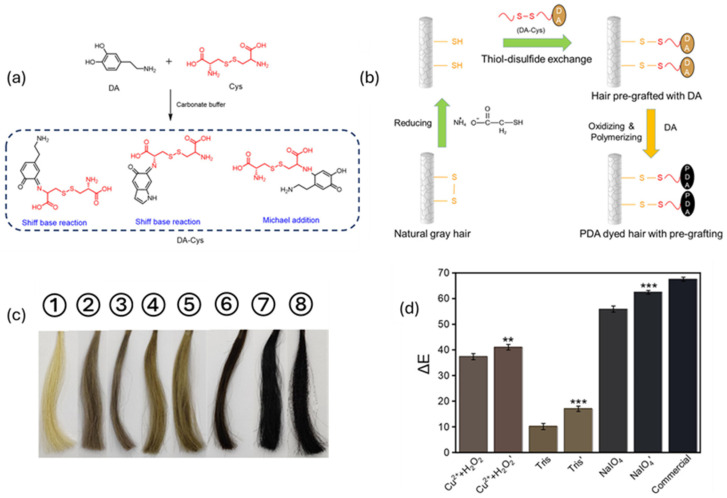
Schematic diagram of proposed method: (**a**) The representative reactions between DA and Cys and (**b**) the PDA hair dyeing process with a pre-grafting treatment. (**c**) Photos and (**d**) ∆E values of (1–8) virgin gray hair and PDA-dyed hair samples dyed in different conditions. Label without “ ′ ” refers to hair without pre-treatment, label with “ ′ ” refers hair with pre-treament. The *, and their number, refers to the *p*-value of ΔE values comparing pre-treated hair and not pre-treated hair (** *p* < 0.01, *** *p* < 0.001) Adapted with permission from ref. [101]. Copyright 2022, American Chemical Society.

The turning point occurred in 2007 when Lee at al. published an article with a meteoric impact in the field [96]. The authors described a facile method to deposit homogeneous layers of PDA, a eumelanin analog, on a wide variety of surfaces, from organic ones to ceramic ones, starting from dopamine (DA). In a short time, DA had become one of the most studied precursors in the synthesis of melanin-like materials. The possibility of grafting PDA on heterogenous substrates in an easy way did not exclude the field of hair dyeing. For example, one of the first attempts was reported by Gao et al., where they described a protocol to attach PDA on hair shafts by coupling the oxidative and grafting properties of metal cations. Briefly, they reported a method to dye hair tresses in 5 min using DA, as a precursor, and the combined effect of CuSO_4_ and H_2_O_2_ [97]. Following the same approach, other researchers took advantage of the great metal-chelating properties of PDA to anchor these materials on hair fibers, like Im et al. [98]. In their work, a PDA solution was prepared through the oxidation of dopamine in a Tris buffer solution. The next step of the protocol was the anchoring of the color on the hair shaft, which was promoted by the addition of metal cations (Fe(II), Fe(III), Al(III), and Cu(II)) into the PDA solution, where hair fibers were previously soaked. Herein, it is interesting to note that, unlike other studies, the SEM images of the surface of the processed hairs appeared smooth in texture, without a clear deposition of nanoparticles on the hair. Hence, it was hypothesized that the major contribution to the pigmentation of tresses was given by the hydrophilic polymeric form of PDA. Indeed, such kinds of morphology have demonstrated a higher amount of catechol units compared to their nanoparticle forms, which should guarantee a better coordination with the metal cations [41]. However, despite the very promising results of the metal-grafting of melanin-like materials on hair cuticles, the use of high concentrations of metal-based ingredients in cosmetic formulations can lead to hampering in terms of regulation and safety for consumers and the environment. Hence, metal-free options were proposed in time. For example, Dong et al. replaced metals with sodium periodate as the oxidizing agent in their formulations [99]. ongoing forward, researchers tried to analyze the effects of other parameters; in fact, despite oxidizers playing a key role in hair dyeing processes, it was discovered that temperature and hair chemistry can have a pivotal role in hair coloration. That was the case for Battistella et al.; they demonstrated that temperature has a crucial role in the adhesion of PDA on hair fibers [100]. Indeed, increasing the working temperature up to 37–40 °C significantly improved the performance of their protocols. Such a boost allowed the application of milder chemical compounds like 3% NH_4_OH, currently involved in commercial hair dye formulations, and in addition to this, the working temperature of 37–40 °C can be easily reached in hair saloons using common devices, such as hair dryers and hairdressing helmets. However, in this case, PDA with a nanoparticle morphology seems to be the material which gives the most major contribution to hair coloration. In the end, we can mention the article of Zheng et al.; their innovative approach follows a pre-functionalization of the hair’s surface to enhance its reactivity with precursors [101]. Here, hair tresses were pre-treated with ammonium thioglycolate to partially reduce disulfide bonds into thiols, followed by immobilizing DA monomers onto the hair’s surface with cysteine (Cys), as schematized in Figure 5. 

**Figure 6:**
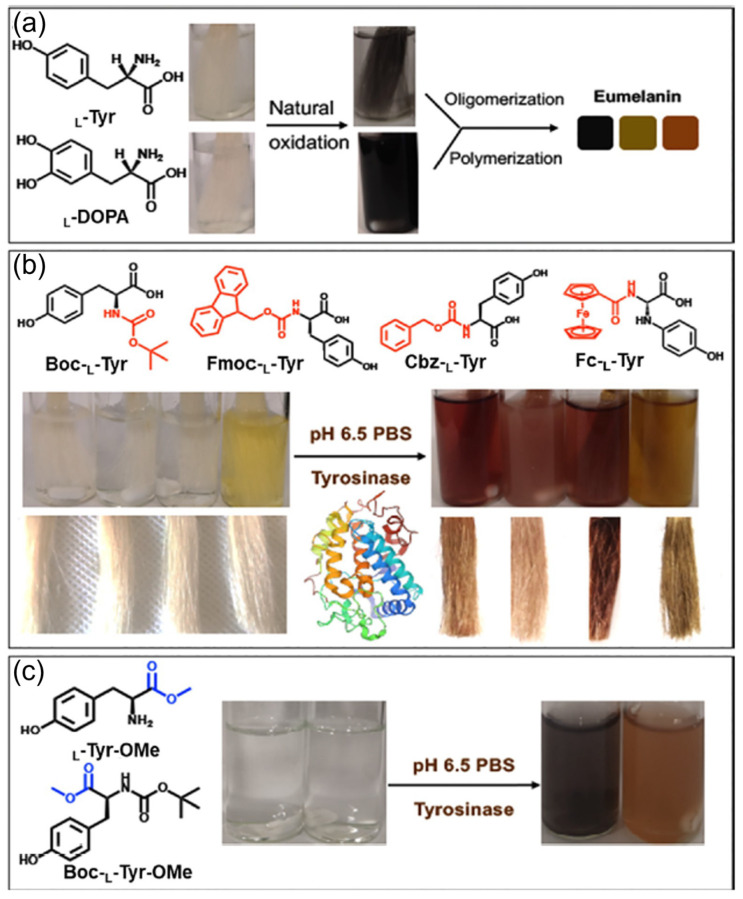
Molecular structure and oxidation process, induced by mushroom tyrosinase (PDB ID: AbPPO4) [105] in phosphate-buffered saline (PBS) of unmodified natural L-Tyr (**a**) and its derivatives after amino and carboxyl modification (**b**,**c**). Adapted with permission from ref. [102]. Copyright 2021, American Chemical Society.

Once the Cys-DA was anchored to the surface of the hair fibers, in was converted into PDA using oxidizers, among which NaIO_4_ demonstrated the most promising outcomes. In summary, according to the proposed protocols, the intended color can be varied by simply changing the involved metal cations and the chemical nature of the oxidizer, while temperature and pre-functionalization of the hair shaft have been demonstrated to tune the deepness of the nuances. As mentioned, between the different strategies proposed to control the biomimetic synthesis and regulation of artificial melanin pigments for hair coloring, the current dominant approach focuses on producing polydopamine coatings, which require an oxidation process often reliant on basic conditions. These conditions not only damage the outer hair layers but also raise concerns regarding biocompatibility and biofriendliness [102]. An alternative approach to precursor oxidation in mild conditions, based on the use of enzymes, was proposed by Sun et al., who effectively achieved PDA deposition through an enzymatic procedure [103]. They demonstrated that in the presence of tyrosine hydroxylase and metal ions, DA could be oxidized into PDA and subsequently self-assembled into nanometer-scale pigments. Metal ions were applied to facilitate self-assembly of PDA and immobilize the pigment NPs onto the hair’s surface. Aqueous solutions of FeCl_3_, FeSO_4_, and CuSO_4_ were investigated. Hair samples treated with and without metal ions, in the presence of DA and tyrosine hydroxylase at room temperature, displayed a significant color change in less than one minute. The type and amount of metal ions and changes in enzyme concentration affect hair darkness, which can be tuned just by altering these parameters. Additionally, hair dyed with PDA shows significant resistance to repeated washing, along with better toughness and softness compared to commercial products. Biocompatibility of the PDA assemblies was confirmed through cytotoxicity testing, further supporting the viability of safe and natural hair pigmentation using this enzymatic approach. A similar strategy was developed by Battistella et al., the main goal of which was producing synthetic melanin coatings that successfully deposit onto human hair through chemoenzymatic oxidation of DA and its precursors or derivatives, avoiding the use of basic conditions [104]. In their study, they avoided uses of metal chelators and, instead, successfully adopted conditions of a neutral pH and 35 °C, which correspond to the optimal reaction parameters for mushroom tyrosinase. These conditions decrease the time needed to obtain effective polymerization and deposition from hours to several minutes and result in minimal perturbation of outer hair layers compared to traditional basic oxidative protocols. This approach allows the use of a variety of monomer substrates, such as tyrosine (L-Tyr), tyramine (TA), and DA, which demonstrate a dyeing ability comparable to commercial dye. Due to their melanin-like nature, the coatings have the potential to absorb radiation and protect fibers from photodamage. While this study effectively demonstrated a fast, mild, and efficient route for producing multifunctional bioinspired hair pigmentation, it did not investigate the long-term durability of the dyeing process or its impact on the mechanical properties of the hair. A limit of these developed processes is their inability to produce a wide range of colors. Shen et al. addressed this challenge by defining a strategy for achieving a broader color spectrum through the enzymatic oxidation of tyrosine derivatives [102]. A series of tyrosine derivatives were selected as precursors for the enzymatic synthesis of melanin-like pigments, such as Boc-L-Tyr, Fmoc-L-Tyr. Cbz-L-Tyr, and Fc-L-Tyr (Figure 6). The study demonstrated that modifying L-Tyr with different protective groups at the N- or C-terminus, along with changing reaction conditions, allowed for regulating the deposition and the assembly of pigments. More precisely, the protected amino groups affected the cyclization of amines and subsequent oxidation process, altering the final color, while carboxyl group functionalization increased steric hindrance, affecting polymerization and leading to lighter shades. Hair dyeing was then carried out at 37 °C and pH 6.5, resulting in successful deposition of the different oxidation pigments on the surface of the hair. The color of the hair could be further tailored by changing the reaction conditions, such as the pH value, enzyme concentration, and tyrosine derivatives concentration. The pigments strongly interacted with the hair’s surface, exhibiting minimal fading after repeated washing. Additionally, the hair’s mechanical properties were not significantly altered by this treatment.

The study also explored the possibility of depositing silver nanoparticles onto the pigment-coated hair through in situ reduction, making these coatings anti-inflammatory and antibacterial. This study, like previous ones, demonstrated the potential of melanin-like nanoparticles as hair dyes, allowing the avoidance of harsh alkaline conditions, thereby reducing hair damage, and aligning perfectly with the modern market’s focus on environmentally friendly solutions.

## 7. Future Perspective

Melanin performs in nature functions such as photoprotection and coloration which are characteristic of some specific cosmetic products. Its use for these applications thus appears to be obvious; nevertheless, although some very good research studies have revealed the incredible properties of natural and artificial melanin NPs for photoprotection and coloration, the real use of these materials in the cosmetic market is still at an embryonic stage. In our opinion, the use of melanin for photoprotection and hair coloration should take into consideration two possible approaches with different advantages and disadvantages that will be shortly discussed.

### 7.1. Use of Preformed Melanin in Cosmetic Products

The main advantage of this approach would be the use of a naturally available source of melanin, and in particular NPs, from the ink of cuttlefish. Other possible “vegan” sources of melanin are available, even if the efficiency of production from these channels is less effective [106,107,108]. An alternative melanin source is the artificial one. Melanin can be prepared in considerable amounts in the laboratory by oxidation/polymerization of molecular precursors and also in mild conditions. In this context, we would like to stress that the most convenient process starts from dopamine, which can be easily oxidized by atmospheric oxygen in mild conditions. As a drawback, dopamine’s cost is too high to be compatible with its use in a large-scale industry like the cosmetic one. Moreover, this molecule is also a neurotransmitter, and the absence of residual dopamine in the final product should be demonstrated. Use of a less-expensive and safer precursor, like tyrosine, has been reported only in the case of enzymatic oxidation [102]. In this case, the cost of the enzyme would make the process not suitable for applications in cosmetics. Hence, naturally extracted melanin is likely to be the best source for cosmetic products based on preformed melanin NPs. Besides this, a major issue of this kind of approach is the formulation of the melanin itself. Melanin NPs have been reported to show a negative Z-potential due to the presence of carboxylates and deprotonated cathecholic groups on their surfaces. As a consequence, melanin NPs are very hydrophilic. Moreover, the photoprotective action of melanin on the skin is still not clearly understood. Indeed, as reported in the recent study of Zamudio Díaz et al. [64], melanin could also induce DNA damage. Indeed, in some cases, it seems to act as a photosensitizer rather than as a photoprotective agent: UV-induced radical species can indeed degrade melanin, leading to species which could transfer their energy to DNA bases. This duality of melanin’s action seems to be strongly dependent on the melanin’s distribution on the epidermis. However, this effect, which could represent a very important issue for melanin’s cosmetic applications, surely needs to be further investigated.

**Preformed melanin NPs for hair coloration**. Human hair is negatively charged and methods for hair coloration typically exploit alkaline pH in order to enhance the hair’s cuticle openings and hence the penetration of the coloring into the hair. Melanin NPs are negatively charged in these conditions (Z-potential about 40 mV for PDA) and hence electrostatic repulsion prevents adhesion of the NPs to the hair. Thus, this approach requires the modification of the surface of the NPs in order to introduce a positive charge. This can be achieved either via covalent or non-covalent functionalization of the NPs’ surfaces. Despite the charge, considering the relatively large size of the melanin NPs (100–200 nm), their actual penetration into the hair cortex is very unlikely and, as demonstrated by experimental results, coloration obtained with this strategy cannot resist washing and it can be only temporary.

**Preformed melanin NPs for photoprotection**. Artificial melanin NPs (PDA) have been demonstrated to be up-taken by human skin cells, keratinocytes, as natural melanosomes. Moreover, these NPs perform in the cell an efficient photoprotective action which combines UV filtration and antioxidant activity. Unfortunately, this protective action could be demonstrated only in vitro and there is no evidence a similar mechanism may operate also in vivo. The external layer of the skin, the epidermis, is an efficient barrier that prevents the penetration of the melanin NPs; hence, more investigation is needed to evaluate the possibility of using preformed melanin NPs as sunscreens. Additionally, the deep dark coloration both natural and artificial melanin makes this material poorly appealing to the cosmetic industry for the formulation of products for photoprotection. This issue may be corrected by tuning the optical properties of melanin either chemically or photochemically. 

### 7.2. Use of Melanin Precursors in Cosmetic Products

#### 7.2.1. Melanin Precursors for Hair Coloration

The use of melanin precursors for hair coloration has been demonstrated to be a winning strategy. Comparing coloration achievable using dopamine and polymerizing it directly on the hair with that obtained by applying preformed PDA NPs, it is clear that the first approach gives a much more intense and stable coloration (i.e., resistant to washing). This difference is due to the fact that dopamine molecules penetrate easily in the hair cortex and they undergo polymerization to form large structures that cannot diffuse out of the hair. The main disadvantage of this approach is that it requires the application to the hair, and hence to the scalp in real use, of dopamine, which is a neuroactive substance and cannot be used in purely cosmetic formulations. Moreover, as mentioned above, dopamine is a quite expensive precursor and this make it not ideal for coloring. Future developments of this approach should take into consideration alternative, cheaper, and less-bioactive precursors such as, e.g., tyrosine. Indeed, use of this molecule as a precursor for hair coloration was considered by the Gianneschi group, but in this case an enzyme, and not just simple oxygen, was necessary to achieve melanin formation and coloration.

#### 7.2.2. Melanin Precursors for Photoprotection

As far as we know, the possible formulation of cosmetic products based on melanin precursor for photoprotection have not been investigated in detail. The most relevant limitation we see in this is that the most investigated precursor of melanin, dopamine, is a neuroactive molecule and is considered a drug by FDA. Hence, its application, even topical, cannot be considered in a simple cosmetic treatment, and classification of a dopamine-based product as a cosmetic formulation would be very unlikely. Moreover, the formation of melanin from molecular precursors requires an oxidative environment that, in the simple case of dopamine, can be simply atmospheric oxygen but that, in other cases, implies the presence of strong chemical oxidants like permanganate or periodate that could damage the skin or living cells. 

### 7.3. Stability of Melanin NPs

Melanin NPs’ stability is another important issue concerning their application in cosmetics. However, this aspect is not solely (or partially) considered when a melanin-based system is developed as a cosmetic. In particular, stability should be considered from several points of view. Indeed, it is not sufficient to assess the stability of melanin NPs in water but also in different media (in organic solvents or in the presence of surfactants or emulsifiers), simulating the characteristic medium for the desired application. Moreover, stability correlated to the potential use of the cosmetic needs also to be assessed. For example, in the case of hair coloration, color maintenance should be established with washing and exposure to light. In sunscreen applications, the resistance to UV-induced photodegradation as well as the resistance to water need to be evaluated. At the moment, in the literature, only a few of these aspects are treated, while they should all have central attention to pave the way for the real application of melanin NPs.

## 8. Conclusions

In conclusion, the potential use of melanin and related materials in cosmetics for photoprotection and hair coloration is surely intriguing, since melanin is already present in living beings and performing these same functions. Recent papers have demonstrated that melanin, and in particular polydopamine, can be indeed used for these purposes in vitro: for photoprotection, in the case of cultured human skin cells, keratinocytes, or for the coloration of cut hair samples. Translation to in vivo applications and to real life use requires further development. The actual photoprotective action of melanin on the skin of living human beings has not been demonstrated yet. Moreover, the use of melanin in cosmetics raises possible toxicity issues related to the size of these materials in the nanometric range, which is considered a possible danger by the present regulations in the cosmetic field, and to the possible presence of highly bioactive molecules like dopamine. As far as hair coloration is concerned, direct formation of melanin into hair represents surely an optimal strategy that is also not too dissimilar from existing coloring approaches. Nevertheless, also in this case, the presence of an expensive and highly neuroactive molecule like dopamine makes it difficult to receive formal approval and the commercialization of any product based on this strategy.

## Figures and Tables

**Table 1:** Nanomaterials allowed in cosmetics according to EU regulations [58].

Category	Nanomaterial	Cosmetic Products
Colorants	Carbon black	Bath/shower products, lip stick, mascara, eye liner, eye pencils, nail varnish, and face masks.
	Titanium dioxide	
	Zinc dioxide	
UV filters	Methylene bisbenzotriazolyl tetramethylbutylphenol	Sun protection products, before- and after-sun products, body care products, self-tanning products, skin lightening products, foundation, hand care products, and lip care products.
	Titanium dioxide	
	Tris-biphenyl triazine	
	Zinc oxide	
Other functions	Alumina	Face masks, nail varnish, nail make-up, bath/shower products, foot care products, mouth wash, shampoo, chemical exfoliation products, soap products, external intimate hygiene products, toothpaste, make-up remover products, eye liner, and eye shadow.
	Colloidal copper	
	Colloidal gold	
	Colloidal platinum	
	Colloidal silver	
	Copper	
	Fullerenes	
	Gold	
	Gold thioethylamino hyaluronic acid	
	Hydrated silica	
	Hydroxyapatite	
	Lithium magnesium sodium silicate	
	Platinum	
	Silica	
	Silica dimethicone silylate	
	Silica dimethyl silylate	
	Silica silylate	
	Silver	
	Sodium magnesium fluorosilicate	
	Sodium magnesium silicate	
	Sodium propoxyhydroxypropyl thiosulfate silica	
	Styrene/acrylate copolymers	

## References

[1] Baghba R., Roshangar L., Jahanban-Esfahlan R., Seidi K., Ebrahimi-Kalan A., Jaymand M., Kolahian S., Javaheri T., Zare P. (2020). Tumor microenvironment complexity and therapeutic implications at a glance. Cell Commun. Signal..

[2] Ferdous Z., Nemmar A. (2020). Health Impact of Silver Nanoparticles: A Review of the Biodistribution and Toxicity Following Various Routes of Exposure. Int. J. Mol. Sci..

[3] Manzano M., Vallet-Regí M. (2020). Mesoporous Silica Nanoparticles for Drug Delivery. Adv. Funct. Mater..

[4] Mitchell M.J., Billingsley M.M., Haley R.M., Wechsler M.E., Peppas N.A., Langer R. (2021). Engineering precision nanoparticles for drug delivery. Nat. Rev. Drug Discov..

[5] Sies H., Jones D.P. (2020). Reactive oxygen species (ROS) as pleiotropic physiological signalling agents. Nat. Rev. Mol. Cell Biol..

[6] Wang Q.Y., Wang Y.P., Ding J.J., Wang C.H., Zhou X.H., Gao W.Q., Huang H.W., Shao F., Liu Z.B. (2020). A bioorthogonal system reveals antitumour immune function of pyroptosis. Nature.

[7] Dordevic L., Arcudi F., Cacioppo M., Prato M. (2022). A multifunctional chemical toolbox to engineer carbon dots for biomedical and energy applications. Nat. Nanotechnol..

[8] Ruan P.C., Liang S.Q., Lu B.G., Fan H.J., Zhou J. (2022). Design Strategies for High-Energy-Density Aqueous Zinc Batteries. Angew. Chem.-Int. Ed..

[9] Zhu Z.X., Jiang T.L., Ali M., Meng Y.H., Jin Y., Cui Y., Chen W. (2022). Rechargeable Batteries for Grid Scale Energy Storage. Chem. Rev..

[10] Mavridi-Printezi A., Menichetti A., Guernelli M., Montalti M. (2021). Extending photocatalysis to the visible and NIR: The molecular strategy. Nanoscale.

[11] Li Z.Z., Wang S.J., Wu J.X., Zhou W. (2022). Recent progress in defective TiO_2_ photocatalysts for energy and environmental applications. Renew. Sustain. Energy Rev..

[12] Yu S.J., Tang H., Zhang D., Wang S.Q., Qiu M.Q., Song G., Fu D., Hu B.W., Wang X.K. (2022). MXenes as emerging nanomaterials in water purification and environmental remediation. Sci. Total Environ..

[13] Guidetti G., Pogna E.A.A., Lombardi L., Tomarchio F., Polishchuk I., Joosten R.R.M., Ianiro A., Soavi G., Sommerdijk N.A.J.M., Friedrich H. (2019). Photocatalytic activity of exfoliated graphite–TiO2 nanoparticle composites. Nanoscale.

[14] Alexandridis P. (1996). Amphiphilic copolymers and their applications. Curr. Opin. Colloid Interface Sci..

[15] Paul B.K., Moulik S.P. (1997). Microemulsions: An overview. J. Dispers. Sci. Technol..

[16] Schilling K., Bradford B., Castelli D., Dufour E., Nash J.F., Pape W., Schulte S., Tooley I., van den Bosch J., Schellauf F. (2010). Human safety review of “nano” titanium dioxide and zinc oxide. Photochem. Photobiol. Sci..

[17] Kango S., Kalia S., Celli A., Njuguna J., Habibi Y., Kumar R. (2013). Surface modification of inorganic nanoparticles for development of organic-inorganic nanocomposites-A review. Prog. Polym. Sci..

[18] McGillicuddy E., Murray I., Kavanagh S., Morrison L., Fogarty A., Cormican M., Dockery P., Prendergast M., Rowan N., Morris D. (2017). Silver nanoparticles in the environment: Sources, detection and ecotoxicology. Sci. Total Environ..

[19] Zhang S.G. (2003). Fabrication of novel biomaterials through molecular self-assembly. Nat. Biotechnol..

[20] Nel A., Xia T., Mädler L., Li N. (2006). Toxic potential of materials at the nanolevel. Science.

[21] De Matteis V., Rinaldi R., Saquib Q., Faisal M., AlKhedhairy A.A., Alatar A.A. (2018). Toxicity Assessment in the Nanoparticle Era. Cellular and Molecular Toxicology of Nanoparticles.

[22] d‘Ischia M., Wakamatsu K., Cicoira F., Di Mauro E., Garcia-Borron J.C., Commo S., Galván I., Ghanem G., Kenzo K., Meredith P. (2015). Melanins and melanogenesis: From pigment cells to human health and technological applications. Pigment. Cell Melanoma Res..

[23] Riley P.A. (1997). Melanin. Int. J. Biochem. Cell Biol..

[24] Huang Y.R., Li Y.W., Hu Z.Y., Yue X.J., Proetto M.T., Jones Y., Gianneschi N.C. (2017). Mimicking Melanosomes: Polydopamine Nanoparticles as Artificial Microparasols. ACS Cent. Sci..

[25] Mavridi-Printezi A., Guernelli M., Menichetti A., Montalti M. (2020). Bio-Applications of Multifunctional Melanin Nanoparticles: From Nanomedicine to Nanocosmetics. Nanomaterials.

[26] Mavridi-Printezi A., Menichetti A., Guernelli M., Montalti M. (2021). The Photophysics and Photochemistry of Melanin- Like Nanomaterials Depend on Morphology and Structure. Chem.—A Eur. J..

[27] Mavridi-Printezi A., Menichetti A., Ferrazzano L., Montalti M. (2022). Reversible Supramolecular Noncovalent Self-Assembly Determines the Optical Properties and the Formation of Melanin-like Nanoparticles. J. Phys. Chem. Lett..

[28] Cao W., Zhou X.H., McCallum N.C., Hu Z.Y., Ni Q.Z., Kapoor U., Heil C.M., Cay K.S., Zand T., Mantanona A.J. (2021). Unraveling the Structure and Function of Melanin through Synthesis. J. Am. Chem. Soc..

[29] Ito S., Wakamatsu K., Sarna T. (2018). Photodegradation of Eumelanin and Pheomelanin and Its Pathophysiological Implications. Photochem. Photobiol..

[30] Simon J.D., Peles D.N. (2010). The Red and the Black. Acc. Chem. Res..

[31] Ito S., Wakamatsu K. (2008). Chemistry of Mixed Melanogenesis—Pivotal Roles of Dopaquinone†. Photochem. Photobiol..

[32] Xu H.W., Zhang Y., Zhang H.T., Zhang Y.R., Xu Q.Q., Lu J.Y., Feng S.P., Luo X.Y., Wang S.L., Zhao Q.F. (2023). Smart polydopamine-based nanoplatforms for biomedical applications: State-of-art and further perspectives. Coord. Chem. Rev..

[33] Jin A.T., Wang Y.T., Lin K.L., Jiang L. (2020). Nanoparticles modified by polydopamine: Working as “drug” carriers. Bioact. Mater..

[34] Ju Y., Liao H.T., Richardson J.J., Guo J.L., Caruso F. (2022). Nanostructured particles assembled from natural building blocks for advanced therapies. Chem. Soc. Rev..

[35] Yang P., Zhu F., Zhang Z.B., Cheng Y.Y., Wang Z., Li Y.W. (2021). Stimuli-responsive polydopamine-based smart materials. Chem. Soc. Rev..

[36] Wang Z., Zou Y., Li Y.W., Cheng Y.Y. (2020). Metal-Containing Polydopamine Nanomaterials: Catalysis, Energy, and Theranostics. Small.

[37] Yang P., Bai W.J., Zou Y., Zhang X.Q., Yang Y.Y., Duan G.G., Wu J.R., Xu Y.T., Li Y.W. (2023). A melanin-inspired robust aerogel for multifunctional water remediation. Mater. Horiz..

[38] McCallum N.C., Son F.A., Clemons T.D., Weigand S.J., Gnanasekaran K., Battistella C., Barnes B.E., Abeyratne-Perera H., Siwicka Z.E., Forman C.J. (2021). Allomelanin: A Biopolymer of Intrinsic Microporosity. J. Am. Chem. Soc..

[39] Zhou X., McCallum N.C., Hu Z., Cao W., Gnanasekaran K., Feng Y., Stoddart J.F., Wang Z., Gianneschi N.C. (2019). Artificial Allomelanin Nanoparticles. ACS Nano.

[40] Meredith P., Powell B.J., Riesz J., Nighswander-Rempel S.P., Pederson M.R., Moore E.G. (2006). Towards structure-property-function relationships for eumelanin. Soft Matter.

[41] Mavridi-Printezi A., Giordani S., Menichetti A., Mordini D., Zattoni A., Roda B., Ferrazzano L., Reschiglian P., Marassi V., Montalti M. (2023). The dual nature of biomimetic melanin. Nanoscale.

[42] Zou Y., Chen X., Yang P., Liang G., Yang Y., Gu Z., Li Y. (2002). Regulating the absorption spectrum of polydopamine. Sci. Adv..

[43] Kohl F.R., Grieco C., Kohler B. (2020). Ultrafast spectral hole burning reveals the distinct chromophores in eumelanin and their common photoresponse. Chem. Sci..

[44] Petropoulos V., Mavridi-Printezi A., Menichetti A., Mordini D., Kabacinski P., Gianneschi N.C., Montalti M., Maiuri M., Cerullo G. (2024). Sub-50 fs Formation of Charge Transfer States Rules the Fate of Photoexcitations in Eumelanin-Like Materials. J. Phys. Chem. Lett..

[45] Grieco C., Kohl F.R., Hanes A.T., Kohler B. (2020). Probing the heterogeneous structure of eumelanin using ultrafast vibrational fingerprinting. Nat. Commun..

[46] Ju K.-Y., Fischer M.C., Warren W.S. (2018). Understanding the Role of Aggregation in the Broad Absorption Bands of Eumelanin. ACS Nano.

[47] Yue Y., Zhao X. (2021). Melanin-Like Nanomedicine in Photothermal Therapy Applications. Int. J. Mol. Sci..

[48] Guernelli M., Bakalis E., Mavridi-Printezi A., Petropoulos V., Cerullo G., Zerbetto F., Montalti M. (2022). Photothermal motion: Effect of low-intensity irradiation on the thermal motion of organic nanoparticles. Nanoscale.

[49] Sklar L.R., Almutawa F., Lim H.W., Hamzavi I. (2013). Effects of ultraviolet radiation, visible light, and infrared radiation on erythema and pigmentation: A review. Photochem. Photobiol. Sci..

[50] Bailey C.G., Nothling M.D., Fillbrook L.L., Vo Y., Beves J.E., McCamey D.R., Stenzel M.H. (2023). Polydopamine as a Visible-Light Photosensitiser for Photoinitiated Polymerisation. Angew. Chem. Int. Ed..

[51] Yang P., Zhang S., Chen X., Liu X., Wang Z., Li Y. (2020). Recent developments in polydopamine fluorescent nanomaterials. Mater. Horiz..

[52] Mavridi-Printezi A., Menichetti A., Mordini D., Amorati R., Montalti M. (2023). Recent Applications of Melanin-like Nanoparticles as Antioxidant Agents. Antioxidants.

[53] Mavridi-Printezi A., Mollica F., Lucernati R., Montalti M., Amorati R. (2023). Insight into the Antioxidant Activity of 1,8-Dihydroxynaphthalene Allomelanin Nanoparticles. Antioxidants.

[54] Salvioni L., Morelli L., Ochoa E., Labra M., Fiandra L., Palugan L., Prosperi D., Colombo M. (2021). The emerging role of nanotechnology in skincare. Adv. Colloid Interface Sci..

[55] McClements D.J. (2012). Nanoemulsions versus microemulsions: Terminology, differences, and similarities. Soft Matter.

[56] Najahi-Missaoui W., Arnold R.D., Cummings B.S. (2021). Safe Nanoparticles: Are We There Yet?. Int. J. Mol. Sci..

[57] Regulation (EC) No 1223/2009 of the European Parliament and of the Council. https://eur-lex.europa.eu/legal-content/EN/TXT/PDF/?uri=CELEX:02009R1223-20180801.

[58] Catalogue of Nanomaterials in Cosmetic Products Placed on The market. https://ec.europa.eu/docsroom/documents/38284.

[59] Inventory of Existing Cosmetic Ingredients in China (IECIC). https://www.chemsafetypro.com/Topics/Cosmetics/China_IECIC_Finder.html.

[60] Cosmetic Products & Ingredients. https://www.fda.gov/cosmetics/cosmetic-products-ingredients.

[61] Regulatory Information for Cosmetics. https://www.canada.ca/en/health-canada/services/consumer-product-safety/cosmetics/regulatory-information.html.

[62] ASEAN Definition of Cosmetics and Illustrative List by Category of Cosmetic Products. https://aseancosmetics.org/uploads/UserFiles/File/TECHNICAL%20DOCUMENTS/Technical%20Documents.pdf.

[63] Nguyen N.T., Fisher D.E. (2019). MITF and UV responses in skin: From pigmentation to addiction. Pigment. Cell Melanoma Res..

[64] Zamudio Díaz D.F., Busch L., Kröger M., Klein A.L., Lohan S.B., Mewes K.R., Vierkotten L., Witzel C., Rohn S., Meinke M.C. (2024). Significance of melanin distribution in the epidermis for the protective effect against UV light. Sci. Rep..

[65] Fajuyigbe D., Douki T., Van Dijk A., Sarkany R.P.E., Young A.R. (2021). Dark cyclobutane pyrimidine dimers are formed in the epidermis of Fitzpatrick skin types I/II and VI in vivo after exposure to solar-simulated radiation. Pigment. Cell Melanoma Res..

[66] Domingues L., Hurbain I., Gilles-Marsens F., Sirés-Campos J., André N., Dewulf M., Romao M., Viaris De Lesegno C., Macé A.-S., Blouin C. (2020). Coupling of melanocyte signaling and mechanics by caveolae is required for human skin pigmentation. Nat. Commun..

[67] Wu X.S., Masedunskas A., Weigert R., Copeland N.G., Jenkins N.A., Hammer J.A. (2012). Melanoregulin regulates a shedding mechanism that drives melanosome transfer from melanocytes to keratinocytes. Proc. Natl. Acad. Sci. USA.

[68] Brenner M., Hearing V.J. (2008). The Protective Role of Melanin Against UV Damage in Human Skin. Photochem. Photobiol..

[69] Mansour O.T., Venero D.A. (2021). Insights into the structure of sunscreen lotions: A small-angle neutron scattering study. RSC Adv..

[70] Santander Ballestín S., Luesma Bartolomé M.J. (2023). Toxicity of Different Chemical Components in Sun Cream Filters and Their Impact on Human Health: A Review. Appl. Sci..

[71] Nitulescu G., Lupuliasa D., Adam-Dima I., Nitulescu G.M. (2023). Ultraviolet Filters for Cosmetic Applications. Cosmetics.

[72] Wang C., Wang D., Dai T., Xu P., Wu P., Zou Y., Yang P., Hu J., Li Y., Cheng Y. (2018). Skin Pigmentation-Inspired Polydopamine Sunscreens. Adv. Funct. Mater..

[73] Biba E. (2014). The sunscreen pill. Nature.

[74] Tolbert S.H., McFadden P.D., Loy D.A. (2016). New Hybrid Organic/Inorganic Polysilsesquioxane-Silica Particles as Sunscreens. ACS Appl. Mater. Interfaces.

[75] Ju E.G., Dong K., Wang Z.Z., Zhang Y., Cao F.F., Chen Z.W., Pu F., Ren J.S., Qu X.G. (2017). Confinement of Reactive Oxygen Species in an Artificial-Enzyme-Based Hollow Structure To Eliminate Adverse Effects of Photocatalysis on UV Filters. Chem.—A Eur. J..

[76] Supanakorn G., Thiramanas R., Mahatnirunkul T., Wongngam Y., Polpanich D. (2022). Polydopamine-Based Nanoparticles for Safe Sunscreen Protection Factor Products with Enhanced Performance. ACS Appl. Nano Mater..

[77] Liberti D., Alfieri M.L., Monti D.M., Panzella L., Napolitano A. (2020). A Melanin-Related Phenolic Polymer with Potent Photoprotective and Antioxidant Activities for Dermo-Cosmetic Applications. Antioxidants.

[78] Li N., Ji X., Mukherjee S., Yang B., Ren Y., Wang C., Chen Y. (2023). A Bioinspired Skin UV Filter with Broadband UV Protection, Photostability, and Resistance to Oxidative Damage. ACS Appl. Mater. Interfaces.

[79] Li N.N., Ji X.H., Wang B.L., Guo Y.L., Wang C.H., Chen Y.S. (2021). Functional composite hydrogels entrapping polydopamine hollow nanoparticles for highly efficient resistance of skin penetration and photoprotection. Mater. Sci. Eng. C-Mater. Biol. Appl..

[80] Abdel-Maksoud G., El-Amin A.R. (2011). A review on the materials used during the mummification processes in ancient Egypt. Mediterr. Archaeol. Archaeom..

[81] Dakin R., Montgomerie R. (2013). Eye for an eyespot: How iridescent plumage ocelli influence peacock mating success. Behav. Ecol..

[82] Xiao M., Li Y.W., Allen M.C., Deheyn D.D., Yue X.J., Zhao J.Z., Gianneschi N.C., Shawkey M.D., Dhinojwala A. (2015). Bio-Inspired Structural Colors Produced *via* Self-Assembly of Synthetic Melanin Nanoparticles. ACS Nano.

[83] McGraw K.J., Safran R.J., Wakamatsu K. (2005). How feather colour reflects its melanin content. Funct. Ecol..

[84] Mundy N.I., Kelly J. (2003). Evolution of a pigmentation gene, the melanocortin-1 receptor, in primates. Am. J. Phys. Anthropol..

[85] Jablonski N.G., Chaplin G. (2017). The colours of humanity: The evolution of pigmentation in the human lineage. Philos. Trans. R. Soc. B-Biol. Sci..

[86] Birngruber C.G., Verhoff M.A. (2012). The color of human hair. Handbook of Hair in Health and Disease.

[87] Itou T., Ito S., Wakamatsu K. (2022). Effects of Aging on Hair Color, Melanosomes, and Melanin Composition in Japanese Males and Their Sex Differences. Int. J. Mol. Sci..

[88] Pandhi D., Khanna D. (2013). Premature graying of hair. Indian J. Dermatol. Venereol. Leprol..

[89] Jo S.K., Lee J.Y., Lee Y., Kim C.D., Lee J.H., Lee Y.H. (2018). Three Streams for the Mechanism of Hair Graying. Ann. Dermatol..

[90] Draelos Z.D. (2006). Sunscreens and hair photoprotection. Dermatol. Clin..

[91] Pointer S. (2005). The Artifice of Beauty: A History and Practical Guide to Perfumes and Cosmetics.

[92] Seo J.A., Bae I.H., Jang W.H., Kim J.H., Bak S.Y., Han S.H., Park Y.H., Lim K.M. (2012). Hydrogen peroxide and monoethanolamine are the key causative ingredients for hair dye-induced dermatitis and hair loss. J. Dermatol. Sci..

[93] He L., Michailidou F., Gahlon H.L., Zeng W.B. (2022). Hair Dye Ingredients and Potential Health Risks from Exposure to Hair Dyeing. Chem. Res. Toxicol..

[94] Brown K., Mayer A., Murphy B., Schultz T., Wolfram L. (1989). Hair coloring by melanin precursors—A novel system for imparting durable yet reversible color effects. J. Soc. Cosmet. Chem..

[95] Brown K.C., Marlowe E., Prota G., Wenke G. (1997). A novel natural-based hair coloring process. J. Soc. Cosmet. Chem..

[96] Lee H., Dellatore S.M., Miller W.M., Messersmith P.B. (2007). Mussel-inspired surface chemistry for multifunctional coatings. Science.

[97] Gao Z.F., Wang X.Y., Gao J.B., Xia F. (2019). Rapid preparation of polydopamine coating as a multifunctional hair dye. RSC Adv..

[98] Im K.M., Kim T.W., Jeon J.R. (2017). Metal-Chelation-Assisted Deposition of Polydopamine on Human Hair: A Ready-to-Use Eumelanin-Based Hair Dyeing Methodology. ACS Biomater. Sci. Eng..

[99] Dong Y.Y., Qiu Y., Gao D., Zhang K.L., Zhou K., Yin H.G., Yi G.Y., Li J., Xia Z.N., Fu Q.F. (2019). Melanin-mimetic multicolor and low-toxicity hair dye. RSC Adv..

[100] Battistella C., McCallum N.C., Gnanasekaran K., Zhou X.H., Caponetti V., Montalti M., Gianneschi N.C. (2020). Mimicking Natural Human Hair Pigmentation with Synthetic Melanin. ACS Cent. Sci..

[101] Zheng C., Huang J., Li T., Wang Y., Jiang J., Zhang X.H., Huang L., Xia B.H., Dong W.F. (2022). Permanent Low-Toxicity Hair Dye Based on Pregrafting Melanin with Cystine. ACS Biomater. Sci. Eng..

[102] Shen Y.H., Liu J.Y., Wang Y.F., Qi W., Su R.X., He Z.M. (2021). Colorful Pigments for Hair Dyeing Based on Enzymatic Oxidation of Tyrosine Derivatives. ACS Appl. Mater. Interfaces.

[103] Sun Y., Wang C.Y., Sun M., Fan Z. (2021). Bioinspired polymeric pigments to mimic natural hair coloring. RSC Adv..

[104] Battistella C., McCallum N.C., Vanthournout B., Forman C.J., Ni Q.Z., La Clair J.J., Burkart M.D., Shawkey M.D., Gianneschi N.C. (2020). Bioinspired Chemoenzymatic Route to Artificial Melanin for Hair Pigmentation. Chem. Mater..

[105] Pretzler M., Bijelic A., Rompel A. (2017). Heterologous expression and characterization of functional mushroom tyrosinase (A*b*PPO4). Sci. Rep..

[106] Yang X., Tang C., Zhao Q., Jia Y., Qin Y., Zhang J. (2023). Melanin: A promising source of functional food ingredient. J. Funct. Foods.

[107] Pralea I.E., Moldovan R.C., Petrache A.M., Ilies M., Heghes S.C., Ielciu I., Nicoara R., Moldovan M., Ene M., Radu M. (2019). From Extraction to Advanced Analytical Methods: The Challenges of Melanin Analysis. Int. J. Mol. Sci..

[108] Guo L., Li W., Gu Z., Wang L., Guo L., Ma S., Li C., Sun J., Han B., Chang J. (2023). Recent Advances and Progress on Melanin: From Source to Application. Int. J. Mol. Sci..

